# Molecular regulation of kidney development: is the answer blowing in the Wnt?

**DOI:** 10.1007/s00467-007-0504-4

**Published:** 2007-06-07

**Authors:** Calli E. Merkel, Courtney M. Karner, Thomas J. Carroll

**Affiliations:** Departments of Internal Medicine (Nephrology) and Molecular Biology, University of Texas Southwestern Medical Center, 5323 Harry Hines Blvd., Dallas, TX 75390-8856 USA

**Keywords:** Renal vesicle, Ureteric bud, β-Catenin, Metanephros

## Abstract

Development of the metanephric kidney is a complicated process regulated by reciprocal signals from the ureteric bud and the metanephric mesenchyme that regulate tubule formation and epithelial branching morphogenesis. Over the past several years, several studies have suggested that Wnt signaling is involved in multiple aspects of normal kidney development as well as injury response and cancer progression. We will review these data here.

## Introduction

During embryonic development, three sets of kidneys form within the mammalian intermediate mesoderm [1]. The three kidney types form in a temporal as well as anterior-to-posterior sequence. The first to form (and the most anterior) is the pronephros. Although in mammals this organ appears to be non-functional from a physiological standpoint, it is essential for the development of other tissues and cell types, including the mesonephric kidney. The mesonephros appears to be physiologically functional in that its tubules are vascularized and it does produce urine; however, this function is completely dispensable for normal embryogenesis in mice. The situation is slightly different in humans, where mesonephric function plays an important role in maintaining amniotic fluid, which is essential for the proper development of other organs such as the lungs [2]. In addition, the mesonephros is required for formation of the male and female reproductive tracts in all vertebrates. The last kidney to form is termed the metanephros, and this organ will become the functional adult kidney in mammals.

The metanephric kidney serves an essential role in tissue homeostasis by regulating the balance of water and electrolytes in the plasma. It also excretes metabolic waste products and regulates the production of certain hormones. As defects in the development of the urinary system constitute some of the most common human birth defects, a better understanding of the genes required for formation of this organ is of great interest. Here, we will review data demonstrating that the Wnt pathway plays critical roles in multiple cellular events that occur during the development of the mammalian kidney.

## Kidney development

**Fig. 1:**
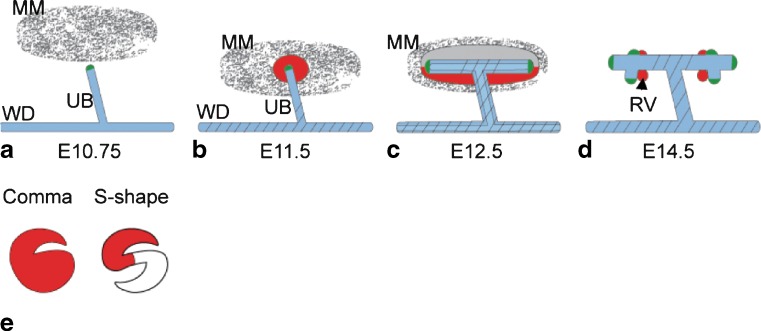
Schematic of kidney development. **a** At E 10.75, the UB forms from the Wolffian duct (*WD*). **b** The UB invades the MM at E 11.5. **c** The UB forms a T-bud, and the MM condenses. **d** Branching morphogenesis of the UB takes place, and the renal vesicles (*RVs*) begin to form. **e** The RVs will then become comma- and S-shaped bodies, and the branching UB forms the collecting duct. The S-shaped body will fuse to the collecting duct and undergo further morphogenesis to become the nephron (not shown). **a**–**e** Expression of Wnts in the developing kidney are indicated as follows: *green* Wnt11, *blue* Wnt9b, *red* Wnt4, *diagonal lines* Wnt7b

Development of the metanephros begins on embryonic day (E) 10 in mice (approximately E 32 in humans), when a caudal portion of the Wolffian duct adjacent to the hindlimbs branches dorsally and invades a population of pre-specified mesenchyme known as the metanephric mesenchyme (MM) (Fig. 1a,b). This epithelial bud, known as the ureteric bud (UB), will continue to branch within the expanding MM throughout the embryonic period and, perhaps, for a short period after birth. Ultimately, the UB derivatives will form the collecting duct system and extra-renal ureter.

Shortly after invasion by the UB, a group of mesenchymal cells within the MM will condense and form a cap around the tips of the UB (Fig. 1c). A subset of cells within the condensate will aggregate and undergo a mesenchymal-to-epithelial transition, forming the renal vesicles (RVs) (Fig. 1d). The majority of the nephron, from the renal corpuscle through the distal tubule, is derived from the RV. The RVs will undergo morphogenesis, forming comma- then S-shaped bodies (Fig. 1e). A capillary will invade the cleft of the proximal part of the S-shaped body and begin to form the glomerulus. The distal portion of the S-shaped body will fuse to the UB, forming one continuous lumen. Non-epithelial portions of the MM will contribute to the smooth muscle, stroma and, perhaps, to the microvasculature of the kidney [3]. The processes of UB branching, RV formation, growth and morphogenesis reiterate themselves during development until, depending on the organism, the kidney takes on its final form and size. In the mouse, this is approximately 1 cm in rostral/caudal length and 10,000–20,000 nephrons, while the average adult human kidney is 10–12 cm in length and contains 500,000–1,000,000 nephrons.

Embryological studies have shown that kidney development depends on inductive interactions between the UB and the MM for the survival, proliferation and differentiation of the MM and the reiterative branching of the UB [4–12]. Identifying the ligands and receptors regulating these processes has been a major emphasis in the field over the past 20 years, and substantial progress has been made.

Several Wnts are expressed in the developing mouse kidney. Subsequent studies using misexpression and functional deletion have shown distinct roles for this pathway in organ formation and disease. In this review, we will cover the known and hypothesized roles for Wnt signaling in development of the mouse kidney.

## Wnt signaling

The Wnts make up a family of secreted glycoproteins that have been implicated in embryonic induction, cell polarity generation, and cell fate specification in metazoan species from hydra to humans [13, 14]. The mammalian genome contains 19 individual Wnt ligands that elicit distinct subcellular events based on the environment in which the signal is received. In the majority of cases, signaling is initiated upon ligand binding to the cysteine-rich, extra-cellular domain of a frizzled (Fz) seven-pass transmembrane receptor and, in some cases, the low-density lipoprotein 5 and 6 co-receptors (*Lrp5* and *Lrp6* in mammals, *arrow* in flies) [15–17]. Upon binding of the ligand to its receptor(s), there are several distinct signal transduction cascades utilized by the Wnts that can be roughly grouped as the canonical/β-catenin pathway and the non-canonical/β-catenin independent pathways.

### The canonical pathway

**Fig. 2:**
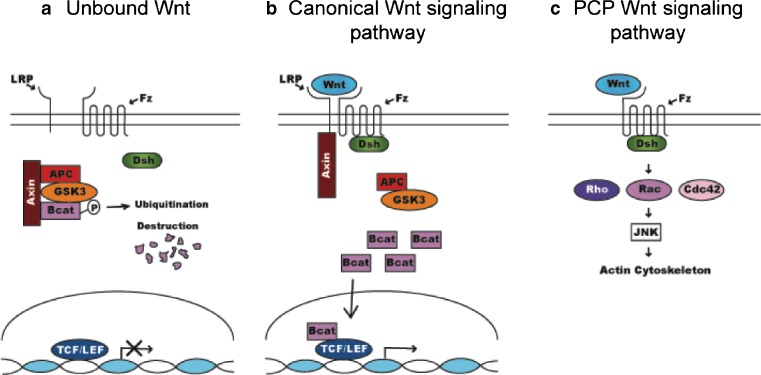
Summary of Wnt signaling. **a** In the absence of bound Wnt ligand, β-catenin is degraded, due to phosphorylation by GSK-3 beta and binding to the destruction complex. **b** In canonical signaling, binding of a Wnt to its Fz receptor and Lrp co-receptor results in inactivation of the destruction complex. This allows β-catenin to accumulate in the cytoplasm and translocate into the nucleus, where it activates transcription of Wnt target genes in cooperation with Lef/Tcf co-factors. **c** In the planar cell polarity (PCP) pathway, Rho, Rac and Cdc42 act downstream of Dsh/Dvl and function to rearrange the actin cytoskeleton and establish cell polarity. In vertebrates, this is thought to be a Wnt-dependent process

The canonical pathway utilizes β-catenin as a transcriptional regulator. In the absence of ligand/receptor interactions, β-catenin levels are regulated by a destruction complex consisting of two scaffolding proteins, axin and adenomatous polyposis coli (APC), and two serine/threonine kinases, glycogen synthase kinase 3 (GSK3) and casein kinase 1 (CK1) (Fig. 2a) [18, 19]. β-Catenin binds to the complex, where it is subsequently phosphorylated by CK1 and GSK3, targeting it for ubiquitination and destruction by the proteasome. The binding of Wnt to Fz activates the cytoplasmic protein disheveled (Dsh/Dvl). Activation of Dvl leads to disruption of the β-catenin destruction complex (through recruitment of axin to the membrane, where it interacts with the cytoplasmic tail of an Lrp) (Fig. 2b). Disruption of the destruction complex results in the accumulation of β-catenin in the cytoplasm. Stabilized, cytoplasmic β-catenin translocates into the nucleus, where it competes with members of the groucho family of co-factors for interactions with the T-cell factor/lymphoid-enhancing factor (TCF/LEF) family of transcription factors. When complexed with groucho and certain other co-factors, the Lef/Tcfs act as transcriptional repressors. It is generally thought that, when complexed with β-catenin, the Lef/Tcfs act as transcriptional activators, although there is an increasing body of data suggesting that certain isoforms of the Lef/Tcfs may act as repressors, even when bound to β-catenin [20].

It should be noted that, in addition to its crucial role in mediating canonical Wnt signaling, β-catenin also plays a role in establishment of the adherens junctions and interacts with the actin cytoskeleton by binding membrane bound E-cadherin and cytoplasmic α-catenin. It has been suggested that Wnt signaling, in part, functions through maintaining a balance between cytoplasmic/nuclear and junctional β-catenin [21, 22].

### The non-canonical pathway

Wnt signaling also appears to trigger β-catenin-independent signaling events. The two most frequently discussed non-canonical/β-catenin-independent pathways are the Ca^2+^-releasing pathway and the planar cell polarity/convergent extension (PCP/CE) pathway. In the Ca^2+^-releasing pathway, Wnt binding stimulates calcium flux and activates calcium-sensitive factors such as protein kinase C (PKC) and calmodulin-dependent protein kinase II (CamKII) [23–25]. This pathway may work in a manner analogous to the Wnt/β-catenin pathway by targeting calcium-responsive transcription factors [26]. There is some disagreement as to whether the calcium pathway signals through Dvl or even the Fz receptors.

Another non-canonical pathway identified is the planar cell polarity (PCP)/convergent extension (CE) pathway, so-named because of the cellular processes the pathway is thought to mediate (Fig. 2c). PCP describes the polarization of cells within the plane of the tissue, while convergent extension is a morphogenetic process that takes place during gastrulation, neurulation and organ formation and describes the intercalation of cells in an epithelial sheet to form a longer and narrower strip of tissue [27, 28].

Components of the PCP/CE pathway were first identified in *Drosophila* mutants and affected the orientation of cuticular bristles, wing hairs and the ommatidia in the eye. Some of the molecules identified had previously been implicated in Wnt signal transduction, including Fz and Dvl, thus leading to the speculation that this may be a Wnt-mediated process. In flies, a molecular readout of PCP is the localization of the proteins strabismus/van gogh (*Stbm/Vang*), and prickle (*Pk*) on the proximal side of the cell and diego (*Dgo*), Fz and Dvl on the distal side. Another protein, flamingo/starry night (*Fmi/Stan*) is also required for PCP, but its localization is not polarized.

Over the past several years, it has become clear that orthologs of the fly PCP/CE genes are essential for various aspects of vertebrate embryogenesis, including gastrulation movements, pelage development and inner-ear formation. Importantly, within the sensory epithelium of the mouse inner ear, Vangl2 (a flamingo homolog), Fz-3 and Fz-6 proteins are asymmetrically localized within the plane of the epithelium, suggesting that the sub-cellular localization, and perhaps function, of at least some of the core PCP components is conserved in mammals [29, 30].

Signaling through the PCP/CE pathway activates alternative downstream elements of Dvl, such as the GTPases Rho, Rac and Cdc42 and the c-Jun N-terminal kinase, JNK [26, 27, 31]. Ultimately, the activation of these molecules orients the cytoskeleton and its associated organelles and protein complexes [32]. This pathway has been implicated in oriented cell migration and oriented cell divisions that affect tissue growth and morphogenesis.

How Wnt signaling mediates PCP is unclear. No mutation of a Wnt ligand has been associated with PCP defects in *Drosophila*, causing some speculation that this is a Wnt-independent event. However, there is evidence of Wnt involvement in PCP establishment in both *C. elegans* and vertebrates [26]. In the *C. elegans* early embryo, the Wnt Mom-2 is both necessary and sufficient for spindle orientation and Fz localization in a transcription-independent manner, and the relative location of the Wnt appears to establish the orientation of the polarity, suggesting that the ligand may play an instructive role in establishing planar cell polarity [33]. Wnt5a and Wnt11 regulate PCP and CE movements during organogenesis and gastrulation in zebrafish and *Xenopus* embryos, although the evidence suggests that, in this system, the ligands may play permissive roles [31, 34, 35].

## Determination of pathway specificity

What determines pathway usage in vivo is still not well understood. Historically, it was thought that pathway specificity was determined by the ligand itself. The Wnts were grouped into canonical or non-canonical classes based on their ability to transform C57MG cells, induce secondary axes in *Xenopus* embryos or induce tubule formation in isolated kidney mesenchyme. Wnts that could act positively in these assays were considered canonical Wnts, while those that could not were considered non-canonical. Although these rough classifications were used for many years, it was soon apparent that not all Wnts could be neatly grouped into one class or the other. Although Wnt1, Wnt3a and Wnt8 always seemed to trigger the canonical event, and Wnt5a always appeared to act non-canonically, other Wnts acted in a canonical manner in some assays and a non-canonical manner in others. For instance, Wnt4 cannot transform C57s or induce a secondary axis in *Xenopus* (putting it in the non-canonical class), but it does induce tubule formation in isolated mouse mesenchyme. Wnt11 does transform C57s but does not induce a secondary axis or induce tubules. Much of this discrepancy could be explained if there was binding specificity between ligands and various receptors and that either affinity for the receptor determined pathway usage, or that different receptors signaled specifically through the canonical or non-canonical pathways. In support of the former hypothesis, He et al. showed that *Wnt5a* was capable of acting canonically (inducing a secondary axis in *Xenopus*) if its mRNA was co-injected with that of *Fz5*. They hypothesized that the inability of Wnt5a to act canonically was due to the absence of its “canonical receptor” from the *Xenopus* early embryo.

Subsequent studies have shown that, in fact, Wnt5a, and other so-called non-canonical Wnts, actively inhibit canonical signaling [36]. In some cases, this ability to repress canonical signaling is mediated by a distinct class of previously orphan, tyrosine kinase-like receptors known as the Rors [37]. Ror2 activity appears to activate Jnk, and active Jnk has been shown to prevent accumulation of nuclear β-catenin [38, 39]. Thus, a potential model is that, if a Ror is present, Wnt5a binds to it and inhibits canonical signaling through activation of Jnk. If Ror2 is absent, Wnt5a can bind to a frizzled receptor and activate canonical signaling.

A further level of regulation is most likely determined by the cytoplasmic environment in which the signal is received. Various intracellular inhibitors of the canonical pathway have been identified, and several of these appear to work on the Dvl protein, acting as switches that divert signaling through the non-canonical pathway [40, 41]. In addition, there are several known co-factors for Lef/Tcf that may compete with β-catenin for binding and thus block canonical pathway activation [20].

## The role of Wnts in kidney development

Analysis of mouse embryos by in situ hybridization has revealed the expression of six Wnts in the developing kidney: Wnt2b, Wnt4, Wnt6, Wnt7b, Wnt9b, and Wnt11 [9, 42–45]. Another eight Wnts have been shown to be present by sequencing of urogenital system cDNAs, with only Wnt5b identified as being specifically located in the kidney. In this section we will cover reported and hypothesized roles for several Wnts that have been implicated in various aspects of kidney development.

### Wnts in renal vesicle formation

**Fig. 3:**
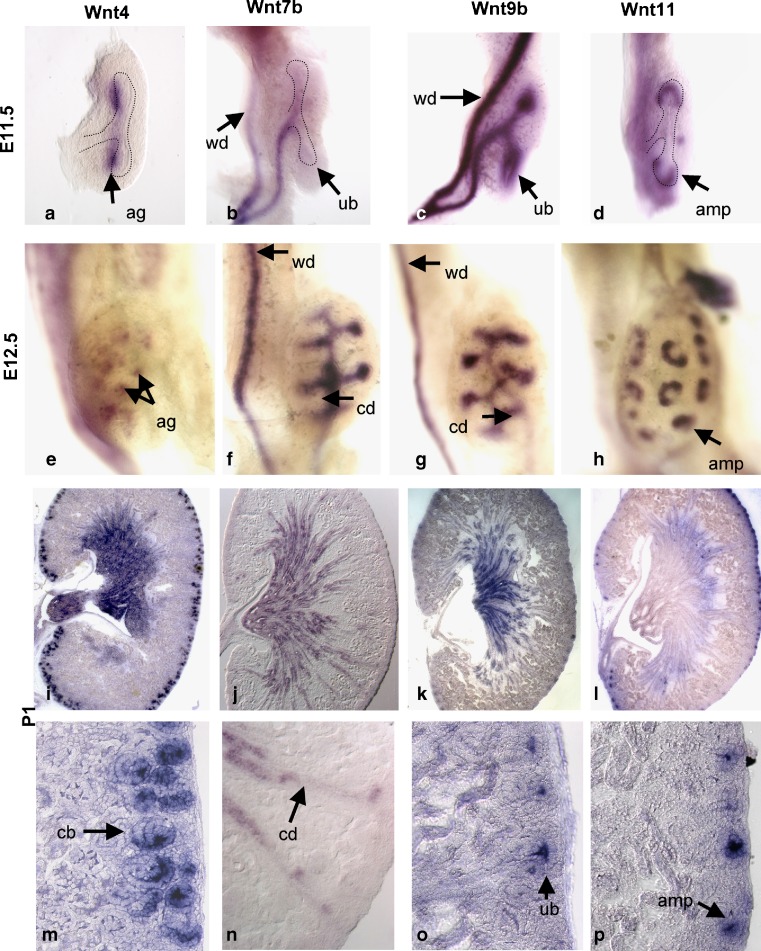
Wnt expression in the developing kidney. The expression pattern of Wnt4 (**a**, **e**, **i**, **m**), Wnt7b (**b**, **f**, **j**, **n**), Wnt9b (**c**, **g**, **k**, **o**), and Wnt11 (**d**, **h**, **i**, **p**) are shown in the developing kidney by whole-mount in situ hybridization at E 11.5 (**a**–**d**) and E 12.5 (**e**–**h**). Expression of the Wnts at P1 is shown by section in situ hybridization (**i**–**p**). **m**–**p** are high-magnification views of the cortex of kidneys shown in **i**–**l**. All hybridizations were performed with previously characterized probes and techniques [42]. Wnt7b P1 images provided by Jing Yu. *ag* aggregate, *wd* Wolffian duct, *ub* ureteric bud, *amp* ureteric bud ampullae, *cd* collecting duct, *cb* comma-shaped bodies

An interest in Wnt signaling in the process of renal vesicle formation arose in 1994, when it was discovered that Wnt1 could substitute for the UB as an inducer of tubulogenesis [46]. Wnt1 is not expressed in the developing kidney, suggesting that it mimics the activity of another Wnt that fulfills the role in vivo. Since this initial observation, it has been discovered that several Wnts are expressed in the developing UB, including Wnt6, 7b, 9b and 11. Both Wnt6 and 9b are expressed throughout the Wolffian ducts and the ureteric bud/ proximal collecting duct system from E 10.5 through birth, although expression levels are lower in the ampullary tips (Fig. 3c,g,k,o and [42, 43]). Wnt7b is weakly expressed in the Wolffian duct and the stalk of the UB but not in the vertical portion or tips of the bud at E 11.5 and E 12.5 (Fig. 3b and f) [42]. At later stages, Wnt7b is expressed in the ureter and distal collecting duct system (Fig. 3j and n). Wnt11 is expressed in the Wolffian duct at E 9.0 [9]. As the UB invades the MM, Wnt11 is confined to the branching tip of the bud, with no expression in the stalk or the cranial Wolffian duct [9]. When the UB undergoes its first bifurcation at E 11.5, Wnt11 expression splits, and Wnt11 is expressed only at the tips of the T bud (Fig. 3d, [9]). Wnt11 continues to be expressed at the distal tips of the ureter from E 12.5 to E 18.5 (Fig. 3h,l,p).

The expression of these ligands in the developing ureteric bud suggested that they could be playing a role in tubule induction. To test the sufficiency of each of these factors to induce tubules, cells expressing the individual ligands were co-cultured with isolated E 11.0 metanephric mesenchyme. Wnt6, Wnt7b and Wnt9b can all induce tubulogenesis, while Wnt11 cannot [7, 42]. Of the “inducing” ureteric bud-expressed ligands, in subsequent genetic studies, only Wnt9b was supported for a role in tubule induction.

*Wnt9b* ^−/−^ mice die within 24 h of birth due to agenesis of the kidneys [42]. In the *Wnt9b* ^−/−^ mutants, the UB invades the MM, but the UB fails to induce the expression of several pre-tubular aggregate markers, including Wnt4, in the adjacent MM. Wnt4 has also been shown to play an essential role in renal vesicle formation [45]. However, unlike Wnt9b, Wnt4 is required in the target cells, the so-called pre-tubular aggregates. *Wnt4* mRNA is expressed in the aggregates as early as E 11 and continues to be expressed in the RVs and the comma- and S-shaped bodies (Fig. 3a,e,i,m). Expression of Wnt4 is lost in wildtype tubules after the S-shaped body fuses to the collecting duct [45].

**Fig. 4:**
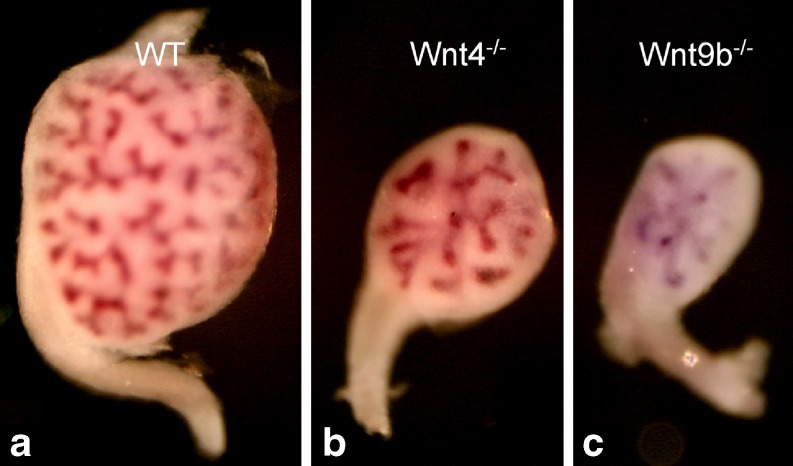
Wnt knockout phenotypes. E 14.5 wild-type (*WT*) (**a**), Wnt4^−/−^ (**b**), and Wnt9b^−/−^ (**c**) kidneys stained with Wnt9b for visualization of the collecting ducts. **b** In Wnt4^−/−^ kidneys, decreased UB branching occurs. **c** In Wnt9b^−/−^ kidneys, there is a more severe defect in UB branching than in Wnt4 mutants

Similar to Wnt9b mutants, *Wnt4* ^−/−^ pups also lack kidneys and die within 24 h of birth. In the *Wnt4* null embryonic kidneys, some branching morphogenesis of the UB takes place, but the mesenchyme fails to convert into epithelial structures by E 12.5 (Fig. 4b and [45]). At E 14.5 a few newly formed RVs can be found in the mutant kidneys, suggesting that another Wnt, perhaps Wnt6 (see below), may be able to compensate for Wnt4 at later stages [3].

Like Wnt9b, Wnt4 is also sufficient to induce RV formation in isolated wildtype MM. Interestingly, Wnt4 can also induce RV formation in Wnt9b mutant MM, suggesting that a major defect underlying the Wnt9b mutant phenotype is the failure to induce expression of Wnt4 [42]. Wnt9b, on the other hand, cannot rescue *Wnt4* mutant mesenchyme, suggesting that the two ligands signal through different receptors and/or branches of the pathway. However, Wnt6 can rescue the *Wnt4* mutants, and *Wnt6* expression levels are increased in *Wnt4* mutants, presenting the possibility that it compensates for Wnt4 at later stages in *Wnt4* null kidneys [43].

The current data indicate that, in wildtype mice, UB-produced Wnt9b is necessary for tubule formation, at least in part through its activation of Wnt4 expression in the adjacent mesenchyme. These data suggest that a continuum of Wnt signaling plays a major role in conversion of mesenchyme to epithelium (Fig. 4c).

The precise mechanism for Wnt function in tubule formation is still not known. It has been suggested that survival and/or proliferation of the mesenchyme is necessary, and perhaps even sufficient, for tubulogenesis. Because a relatively normal mesenchymal population is maintained in Wnt9b (and Wnt4) mutant kidneys through at least E 14.5 and there is no significant change in the rate of apoptosis in E 11.5 Wnt9b mutants, a defect in survival in either of these mutants seems highly unlikely ([42] and data not shown). Although certain survival factors, such as Fgf8, are not expressed in Wnt9b mutant mesenchyme, this is most likely a secondary consequence of a failure to induce pre-tubular aggregate formation and most likely does not play a causal role in the phenotype. In fact, the co-culturing of Wnt9b mutant mesenchyme with recombinant Fgf8 protein does not induce tubule formation or Wnt4 expression (our unpublished observations).

Whether defects in cell proliferation play a role in the *Wnt9b* mutant phenotype is less clear. Although mesenchymal cells in mutant kidneys survive, they do not appear to increase in number. Thus, it is possible that the failure to form tubules is secondarily caused by a failure to reach some critical cell number. However, the mutant metanephric anlage expresses a number of genes associated with its specification and it is of normal size at E 11.0, even though it does not express Wnt4 or Pax8. The only apparent defect at E 11.0 is the failure to induce pre-tubular aggregate markers, suggesting that the mutant phenotype manifests itself prior to any changes in cell number [42]. The simplest explanation for the phenotype is that Wnt9b (and Wnt4) are directly involved in the process of mesenchymal-to-epithelial transition and that this secondarily affects cell proliferation but not cell survival.

There are several ways in which Wnts could play a direct role in mesenchymal-to-epithelial transition. One possibility is that Wnt signaling directly regulates cell adhesion. Such a role has been suggested for Wnt4, whose over-expression results in an increase in the expression of the cell adhesion molecule E-cadherin [47]. Alternatively, Wnt signaling could be regulating other sub-cellular processes, such as cell polarization or vesicular trafficking, that are indirectly necessary for cell adhesion and epithelium formation. A better understanding of the role of Wnt signaling in tubule formation will require a more detailed molecular and cellular analysis of Wnt9b and Wnt4 mutants.

Another question that remains is how Wnt signaling relates to the activity of other factors that appear to be sufficient for kidney tubule formation, including the leukemia inhibitory factor (Lif) and transforming growth factor beta (TGFβ) [48, 49]. Previous studies have shown that, similarly to Wnt9b and Wnt4, both of these factors weakly induce canonical Wnt signaling in isolated mesenchyme. Therefore, it is possible that Lif (or other members of the Il-6 family of cytokines) and/or Tgfβ signaling function upstream of, or in some way mimics, Wnt activity in the mesenchyme. How this relates to the normal in vivo situation is unclear, as genetic analysis has so far not revealed a direct role for either of these signaling pathways in kidney tubule formation.

Another possibility is that Lif and/or TGFβ function by inducing survival and/or proliferation in cells that have already been weakly induced and that this is sufficient to trigger tubulogenesis. Yang et al. showed that isolated rat mesenchymes already expressed Wnt4 prior to their treatment with Lif, suggesting that Lif was either necessary to maintain the Wnt4 expressing cells in the absence of the bud or was working through a parallel pathway that is required along with Wnt signaling [50]. The fact that Wnt4 expression in the rat kidney appears to be independent of the ureteric bud may partially explain why Lif is sufficient to induce tubulogenesis in the rat but not in the mouse, where Wnt4 appears to be dependent on a signal from the bud. It is interesting to note that canonical Wnt signaling appears to be sufficient to induce tubulogenesis in mesenchyme isolated from mouse mutants that do not form a caudal Wolffian duct or ureteric bud (*Gata3* and *Gdnf* mutants, respectively) and, therefore, have never been exposed to any other duct/bud derived signals [51]. These data are suggestive that canonical Wnt signaling acts alone in tubule formation and that other sufficient factors are either acting through the Wnt pathway or through some other mechanism.

### The role of Wnt signaling in branching morphogenesis

Several Wnts are expressed in or around the developing ureteric bud. As mentioned, the expression patterns of Wnt6, Wnt7b, Wnt9b and Wnt11 are all consistent with roles in development of this structure. In addition, at later stages of development, Wnt4 and Wnt11 are both expressed in the medullary stroma, possibly signaling back to the adjacent collecting ducts, although it appears that, at least for Wnt4, the medullary expression is actually mediating the formation of smooth muscle cells [52]. However, for Wnt9b and Wnt11, the hypothesized roles in ureteric bud formation are supported by genetics.

The *Wnt11* mutant phenotype in mice is variably penetrant, with some mutants dying in utero and others dying shortly after birth [53]. In null embryos that survive until birth, the kidneys are histologically normal but are smaller, with 36% fewer glomeruli than in their wild-type littermates. *Gdnf* expression is significantly down-regulated in the mutant mesenchyme. GDNF is the ligand for the Ret receptor tyrosine kinase, which is expressed in the ureteric bud tips. Both Gdnf and Ret are required for normal branching of the UB [54, 55]. When *Ret* ^+/−^ mice are crossed with *Wnt11* ^+/−^ mice, the resulting kidneys of the double heterozygotes are 52% smaller than those of wildtype. *Ret* ^+/−^;*Wnt11* ^−/−^ mice have kidneys 67% the size of those of *Ret* ^+/−^;*Wnt* ^+/−^ mice. These studies indicate that Wnt11 acts in parallel with the Ret/Gdnf pathway to regulate branching of the ureter [53].

In addition to defects in formation of the renal vesicles, Wnt9b mutants also have defects in branching of the ureteric bud [42]. Although the UB undergoes the first bifurcation normally, branching after the T stage is disrupted (Compare Fig. 4a and c). In the *Wnt9b* ^−/−^ mutants, expression of the branching regulators Wnt11 and GDNF is down-regulated prior to the appearance of any morphological signs of branching defects. In addition, the ureteric buds of *Wnt9b* mutants branch significantly less than those of Wnt4 mutants, suggesting that the phenotype is not a secondary effect from failure to form the renal vesicles but instead a direct role for Wnt9b in regulation of secondary branching of the ureteric bud (Fig. 4b). As discussed above, Wnt11 and the Ret/Gdnf pathways normally regulate UB branching. At this point it is unclear whether the cellular target of Wnt9b in branching is the Gdnf-expressing mesenchyme or the Wnt11-expressing ureteric bud.

Signals from the mesenchyme are necessary for the survival and branching of the ureteric bud. Treatment of isolated ureteric buds with the Wnt agonist LiCl sustains branching of isolated ureteric buds. Although numerous Wnts that are expressed in and around the developing bud could mediate this phenotype, Wnt2b appears to be sufficient to fulfill this role. Wnt2b is weakly expressed from E 11.5 to E 13.5 in the perinephric cells surrounding the differentiating mesenchyme [44]. The co-culturing of cells expressing this gene with isolated mesenchyme has no effect; however, co-culture with isolated ureteric buds leads to survival and branching of the bud epithelium. These data suggest that Wnt2b may function to support the initiation of branching of the UB, although there are no genetic data to support this hypothesis.

## Pathway usage in the kidney: canonical or non-canonical?

As mentioned, the branch of the pathway utilized by an individual ligand is dependent on the cellular environment in which the signal is received. The specific pathway used by each of the Wnts involved in the development of the metanephric kidney is unknown. In fact, examination of the expression of pathway components and target genes suggests that the canonical, PCP and calcium branches are all active during metanephric kidney development [7, 56–59]. The challenge now is identifying the ligands that mediate signaling through each branch and the cellular processes they regulate.

It was generally thought that the canonical pathway was involved in tubule induction and the non-canonical pathway in ureteric bud branching. This was based on the observations that stabilization of β-catenin in the mesenchyme and co-culture of mesenchyme with “canonical” Wnts, such as Wnt1 and Wnt3a, induced tubule formation, while mutation of “non-canonical” Wnts, like Wnt11, led to branching defects. This classification also seemed to fit with the established roles for canonical signaling in cell proliferation and adhesion and non-canonical signaling in cell movement/migration. However, recent data suggest that the situation is significantly more complex and that interplay between both branches of the pathway may be necessary for normal renal development.

### Pathway usage in renal vesicle formation

**Fig. 5:**
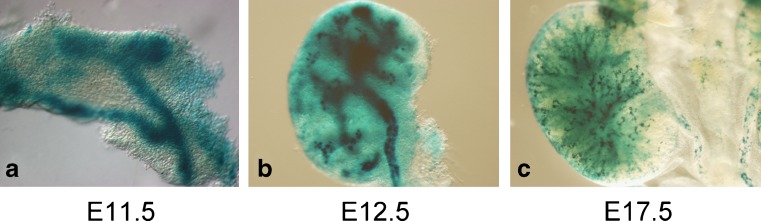
Canonical Wnt signaling in the developing urogenital system. β-galactosidase staining shows canonical Wnt signaling in the developing kidneys of Bat-gal mice. **a** Staining shows activated β-catenin in the Wolffian duct and UB at E 11.5. At E 12.5 (**b**) and E 17.5 (**c**), canonical Wnt signaling is found at high levels in the developing collecting ducts and, perhaps, at lower levels in the MM

Studies making use of a canonical pathway reporter in mice, in which multimerized Lef/Tcf binding sites control expression of the β-galactosidase reporter (the so-called Bat-Gal mouse), as well as examination of the mRNA for a general β-catenin target gene, Axin2, showed little, if any, β-catenin activity in the mesenchyme, while there was high activity in the UB and collecting ducts [60–62] (Fig. 5). These observations suggested that canonical signaling was not directly involved in development of the mesenchyme or the renal vesicles. In fact, work performed by Osafune et al. suggested that the PCP pathway was required in the mesenchyme downstream of Wnt4 during tubule formation and that canonical signaling actually inhibited Wnt4 activity [56]. This was consistent with results obtained by Cai et al., showing that both Wnt4 and Wnt11 could stimulate Jnk activity and that this activated Pax-2, a transcription factor required for multiple aspects of kidney development [63].

These results were seemingly in direct opposition to those in the previous studies on canonical pathway activity in tubule formation. This contradiction could be rectified if one assumed that Wnt9b acted in a canonical manner to stimulate Wnt4 expression, which subsequently induced tubulogenesis through the PCP pathway. However, studies using Madin–Darby canine kidney (MDCK) cells have shown that Wnt4 can activate a β-catenin/LEF/TCF luciferase reporter and Wnt4 can stabilize β-catenin in isolated MM, suggesting that it signals through the canonical branch [42, 57]. Therefore, an alternative model is that the canonical Wnt signaling pathway, mediated by Wnt9b and/or Wnt4, is involved in the early stages of nephrogenesis, driving cell adhesion and/or cell proliferation (This hypothesis supposes that axin2 and Bat-Gal are not faithful reporters of canonical pathway activity in the MM.). Once the pre-tubular aggregates or renal vesicles have formed, Wnt signaling is shifted, through an unknown mechanism, to a non-canonical role, mediating tubule growth/extension. This model suggests that a fine balance between canonical and non-canonical signaling must exist in the developing kidney.

### Pathway usage during branching morphogenesis

Examination of both Bat-Gal and axin2 expression indicates high levels of canonical activity in the branching tips of the ureteric bud and lower levels in the stalk, suggesting that this branch of the pathway may play an important role in ureteric bud/collecting duct development. This fits with the data showing that lithium, an agonist of the canonical pathway, supports branching of isolated buds [44]. What exactly the canonical pathway is doing in the ureteric bud is unknown, and a better understanding may have to wait for tissue-specific ablation studies. One potential role is mediation of the adhesive state of cells in the bud tips. In other models of branching morphogenesis, it has been suggested that the epithelia reverts to a mesenchymal state during branching [64]. Canonical signaling could be mediating such a transition in the kidney, although this would be contrary to other data, where a loss of β-catenin is correlated with epithelial-to-mesenchymal transition [65]. It is possible that Wnt signaling is simply mediating differential adhesiveness between the tips and the stalk, perhaps through regulating the expression or activity of a cell adhesion molecule. Interestingly, the *L1Cam* gene has been identified as a direct transcriptional target of β-catenin, and *L1Cam* has been implicated in mediating normal branching of the UB [66, 67]. Another possibility is that canonical signaling could be maintaining the cells of the ureteric bud in a precursor/undifferentiated state, allowing them to continue dividing and/or branching. Such a role has previously been established for canonical signaling in multiple tissues [68–71].

The ligand mediating canonical signaling in the bud tips is unknown. It could be one of the mesenchymally expressed Wnts or one of the bud-expressed Wnts. Candidates for a mesenchymally expressed Wnt include Wnt2b acting redundantly with another Wnt such as Wnt4 (our unpublished observations rule out a role for mesenchymally expressed Wnt4 alone). Wnt9b or Wnt11 would seem to be good candidates for the bud-expressed Wnts, given their expression patterns and their role in branching morphogenesis, but neither appears to be necessary for axin2 or Bat-gal expression on its own (our unpublished observations). A third possibility is that reporter activation is Wnt independent. Recent data suggest that a number of non-Wnt factors are able to activate the β-catenin pathway, including the secreted molecules norrin, dickopf2, Sfrp1 and the R-spondins [72–76]. Although several of these molecules are expressed in the embryonic kidney, their roles in the development of this organ are unclear, although sFrp-1 has been suggested to have an inhibitory role in tubule formation [77].

There is no direct evidence indicating a role for the non-canonical pathway in the development of the ureteric bud/collecting duct system. As mentioned, in the Bat-Gal mouse β-galactosidase is expressed throughout the bud/ducts through birth. If this reporter represents a faithful readout of pathway activity and if only one pathway is activated in a specific cell type at a particular point in time, then one can conclude that the non-canonical pathway is not playing a major role in the prenatal development of the collecting duct system. However, if axin2 transcripts represent a more faithful readout, then the canonical pathway is not active in the distal collecting ducts, and it is possible that any Wnts expressed in or around this tissue (including Wnt6, Wnt7b and Wnt9b) or the Wnts expressed in the medullary stroma (Wnt4 and Wnt11) may be signaling to the distal collecting ducts through the non-canonical pathway. However, conclusive proof of the involvement of the non-canonical branch will require identification of the receptors for each of the kidney-expressed Wnts as well as a more careful examination of the expression and functional roles of the canonical and non-canonical pathway determinants during renal development. It is tempting to speculate that the processes of convergent extension and/or planar cell polarity are involved in the directional growth of the collecting ducts, and evidence will be presented below suggesting that this is indeed the case.

### Kidney tubule formation and growth; a balancing act?

For years it has been known that improper regulation of the canonical Wnt pathway correlated with and caused various human diseases, including cancers. More recently it has been suggested that a fine balance between the canonical and non-canonical branches of the Wnt pathway is essential for the development and homeostasis of multiple tissues, including the kidney. One of the clearest examples of such a balance comes from functional analysis of the inversin (*Inv*) gene in mice. *Inv* encodes an ankyrin repeat domain encoding protein that has homology to diversin, the vertebrate ortholog of the fly PCP protein, diego. Mutations in *Inv* cause nephronophthisis type II (NPNII), an autosomal recessive disorder characterized by extensive renal cysts [78]. Mutation of *inv* in mice results in renal cysts and *situs inversus* [79].

Studies performed by Simons et al. suggest that inversin plays an important role in regulating Wnt signaling and the choice between the canonical and non-canonical signaling pathways during kidney development [78]. Misexpression of inversin in cultured cells resulted in reduced levels of stabilized cytoplasmic β-catenin. Inv and Dvl physically interacted, and co-expression of *Inv* mRNA repressed the formation of secondary body axes that were induced by Dvl (but not by β-catenin) in *Xenopus* embryos, suggesting that Inv binds to and inhibits the ability of Dvl to mediate canonical Wnt signaling [78]. This study also showed that morpholino-mediated knockdown of *Inv* in zebrafish resulted in increased expression of canonical Wnt targets, and defects in CE movements, a process dependent on non-canonical Wnt signaling [78]. Thus, Inv appears to promote a switch from the canonical pathway to the non-canonical pathway. In *Inv* mutants, the canonical pathway is enhanced, while the non-canonical pathway is abrogated, leading to cyst formation.

It appears that perturbation of the Wnt pathway may play a general role in kidney cystogenesis. Increased levels of β-catenin are strongly correlated with cyst formation in humans and over-expression of β-catenin or c-Myc, a downstream target of β-catenin, in mice gives rise to cystic kidneys [80–87]. Further, deletion of the β-catenin destruction complex protein APC from the kidney epithelia results in increased nuclear β-catenin and formation of cystic tubules [80]. Presumably, over-expression of β-catenin results in increased cell proliferation and perhaps other defects in differentiation, cell adhesion or polarity that contribute to cyst formation.

As the canonical and non-canonical pathways are antagonistic toward each other, over-expression of β-catenin should also antagonize the non-canonical pathway (although this has not been shown in the kidney). Planar cell polarity/convergent extension processes have been shown to be involved in orienting the plane of cell division and/or cell intercalation during the growth of organs such as the heart and the pancreas [27]. Defects in either of these two processes could contribute to the formation of cystic tubules. For example, when the orientation of cell division is randomized by mutation of the PCP gene dachsous, the shape of the *Drosophila* wing becomes shorter and wider [88]. By extrapolation, a change in the orientation of cell division during kidney growth could lead to an increase in tubule diameter, perhaps at the expense of tubule length, and thus contribute to cyst formation. Recently, just such an observation was made in the Hnf1β mutant mouse and *pck* rat models of polycystic kidney disease (PKD). Fischer et al. showed that the orientation of cell division is randomized in these mutants prior to cyst formation, suggesting a causal role in cystogenesis [89]. Although neither of these genes has been directly tied to non-canonical Wnt signaling, defects in the planar cell polarity branch of the pathway would presumably lead to similar defects in the orientation of cell division that would contribute to the cystic phenotype.

Another process shown to be dependent upon planar cell polarity that could contribute to cystogenesis is the intercalation of cells during convergent extension movements [34, 90–92]. Removal of Dvl, Vangl and prickle orthologs results in convergent extension defects during neurulation, cochlear formation and gastrulation, indicating that PCP signaling is required for these morphogenetic movements in vertebrates [93–99]. Cell intercalation has not been demonstrated during the development of the mouse kidney tubules although it does occur during the development of the fly Malpighian tubules [100]. So, it seems plausible that defects in PCP signaling could result in defects in the orientation of cell division and, possibly, in the intercalation of cells during kidney growth, resulting in tubules with an increased diameter. It is currently unclear whether a defect in either branch of the Wnt pathway (activation of the canonical or inactivation of the non-canonical) alone would be sufficient to cause cyst formation or, as the two are mutually antagonistic, whether it is even possible to get one without the other.

Given the extensive data implicating mis-regulation of the Wnt pathway in human disease, there has been intense investigation into the molecular nature of the factors necessary for regulation in vivo. Some of this regulation occurs at the level of the ligand, affecting its ability to interact with its receptors or co-receptors [101]. Such antagonists include the secreted frizzled-related proteins (sFRPs), the Wnt inhibitory factors (Wifs), and the dickkopfs (Dkk) [101]. In the case of the sFRPs, there is some evidence that they play a role in the development of the kidney. Both sFRP-1 and sFRP-2 are expressed in the developing kidney but in distinct patterns. While sFRP-2 expression overlaps the expression of Wnt4 in the condensed mesenchyme and epithelial bodies, sFRP-1 expression is limited to the interstitial mesenchyme [77, 102]. In explant studies, rat metanephroi were treated with sFRP-1, sFRP-2, or both [77]. sFRP-1 exhibited an inhibitory effect on the formation of tubules, while sFRP-2 treatment had no effect. However, when sFRP-2 was combined with sFRP-1, the inhibitory effect of sFRP-1 was decreased. These results suggest that sFRP-2 may function to promote Wnt4 activity (possibly by repressing Wnt9b?), while sFRP-1 assures that tubulogenesis is restricted to the aggregate mesenchyme. sFRP-2 appears to be a target of Wnt-4 activity, as expression is ablated in Wnt4 mutants [77]. How these results relate to the in vivo situation is unclear. Recent studies indicate that sFrp1 and 2 play redundant roles during embryogenesis [103]. Embryos lacking both genes die in utero with anterior–posterior patterning defects, a phenotype that is similar to that of Wnt5a null embryos. Wnt5a does not interact with the sFrps and is generally considered a non-canonical Wnt. These data lend support to the hypothesis that repression of the canonical pathway is necessary for activation of the non-canonical pathway. At this point, there has been no published characterization of the kidneys in the sFRP double mutants.

A second type of regulation appears to be through the production of intercellular antagonists of the signal transduction pathways. As one might expect, several proteins have recently been identified that appear to interact with Dvl to regulate pathway or branch utilization, including the previously discussed Inv, naked cuticle (*nkd*), daple (*Dpl*) and dapper/frodo (*dact/frd*) proteins, although, with the exception of Inv, no role in kidney development has been established for any of these factors [40, 41, 78, 104]. Although the *Inv* mutant kidneys are slightly smaller than normal and cystic ones, much of early nephrogenesis occurs normally. This suggests that either the shift from one branch to the other is not essential during early embryonic stages or that there is another factor that fulfills this role at earlier time points.

And, finally, there are several proteins that affect the ability of β-catenin to function as a transcriptional activator including groucho, kaiso, cited1 and specific isoforms of the Lef/Tcfs [20, 105]. Outside of cited1, there is little known of the expression of these genes during kidney development. However, cited1 is expressed in the condensed kidney mesenchyme, and over-expression of this gene in cultured kidneys blocks tubule formation, suggesting that inhibition of β-catenin activity does play an important role in tubulogenesis.

### Summary and future of the field

Within the past several years we have made tremendous advances in our understanding of the molecular regulation of kidney development, and the Wnt pathway appears to play multiple crucial roles. However, we are still a very long way from comprehending the molecular and cellular function of this pathway during nephrogenesis. In fact, a number of very basic questions remain unanswered.

Much of our current thinking on the role of Wnts is based on mRNA expression and knockout phenotypes. However, recent studies suggest that, in addition to regulating the transcription of the Wnt mRNA, pathway activity may be regulated at the level of ligand secretion. Studies performed on flies and worms have indicated that secretion of Wnts from the producing cells is regulated and that this level of regulation may be conserved in mammals [106–108]. Thus, mRNA expression, and even protein expression, do not equate to the production of active, secreted ligand. A more detailed analysis of the expression of Wnt pathway components and targets within the developing kidney should reveal a great deal about the cellular targets of the Wnt signals. It would also be extremely helpful to know the individual frizzled receptors for each of the ligands. Active Wnt proteins have been difficult to purify, which has hampered the identification of receptor ligand pairs. Nor has genetic analysis been of use, as none of the frizzleds ablated in mice has shown a mutant phenotype in the kidney. Until we gather more information on cellular targets and/or receptors, the precise role of each ligand will remain unclear.

Another unanswered question is the role of some of the alternative pathway components during kidney development. In situ hybridization analysis has shown the expression of the canonical ligands R-spondin1 and R-spondin3 in the peripheral metanephric mesenchyme or stroma [109]. What role are these factors playing in kidney development and how does it relate to the function of the traditional ligands? The non-canonical receptors, Ror1 and Ror2, are also both expressed in the developing kidney [110, 111]. What are the ligands for these molecules and once again, what are their roles?

A final unexplored frontier is the role for Wnts in patterning or maintaining the kidney. Wnt9b is necessary for MET but its mRNA continues to be expressed long after the last tubules have been induced [112]. Wnt4 is also necessary for mesenchymal–epithelial transition (MET), but its mRNA continues to be expressed in a polarized fashion within the comma- and S-shaped bodies [45]. Do either or both of these factors play additional roles in the growth, patterning or maintenance of the kidney epithelia? Until recently, due to the nature of germline knockouts, these questions were difficult, if not impossible, to answer unequivocally. However, the recent advances in techniques allowing temporal and spatial ablation of gene products now make addressing these questions feasible. Although we have already learned a great deal about the role of this important family of molecules in the development of the kidney, the near future almost certainly promises to reveal additional roles in kidney growth, patterning, differentiation and maintenance.
